# Effects of ultra‐high pressure on the morphological and physicochemical properties of lily starch

**DOI:** 10.1002/fsn3.2060

**Published:** 2020-12-24

**Authors:** Dali Zhang, Haishan Xu, Bing Jiang, Xinyu Wang, Lvzhu Yang, Yang Shan, Shenghua Ding

**Affiliations:** ^1^ Longping Branch Graduate School Hunan University Changsha China; ^2^ Hunan Agricultural Product Processing Institute Hunan Academy of Agricultural Sciences Hunan Provincial Key Laboratory for Fruits and Vegetables Storage Processing and Quality Safety Changsha China; ^3^ Hunan Province International Joint Lab on Fruits & Vegetables Processing, Quality and Safety Changsha China

**Keywords:** gelatinization, lily, physical modification, starch, ultra‐high pressure

## Abstract

In this study, starch extracted from lily bulbs were modified using an ultra‐high pressure (UHP) treatment at six different pressure levels (100, 200, 300, 400, 500, and 600 MPa). The effects of UHP treatment on the physicochemical and morphological properties of lily starch were investigated. The morphological observation revealed that UHP treatment led to particle expansion and aggregation. Compared with the native and lily starch treated at 100–500 MPa, the lily starch treated at 600 MPa exhibited almost completely disrupted morphology and a larger particle size, indicating nearly complete gelatinization of the starch. The relative crystallinity of the UHP‐treated starch remarkably reduced. Gelatinization temperatures via differential scanning calorimetry decreased with increasing pressure. The rapid viscoanalyzer results revealed that the lily starch treated with UHP at 600 MPa showed low values of peak viscosity, trough viscosity, breakdown, final viscosity, and setback. These results indicated that UHP was an effective physical modification method for lily starch, UHP treatment (600 MPa, 30 min) caused nearly complete gelatinization of lily starch, and lily starch modified using UHP might expand the application of lily in the food field.

## Graphical abstract

In this study, starch extracted from lily bulbs were modified using an ultra‐high pressure (UHP) treatment at six different pressure levels (100, 200, 300, 400, 500, and 600 MPa). The results indicated that UHP was an effective physical modification method for lily starch, UHP treatment (600 MPa, 30 min) caused complete gelatinisation of lily starch, and lily starch modified using UHP might expand the application of lily in the food field.

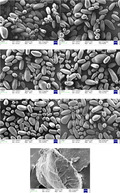

## 1 INTRODUCTION

Lily belongs to the genus *Lilium* of the family Liliaceae, and it is widely distributed in the cold temperate zone of the northern hemisphere; China is one of the major distribution areas of lily plants, with approximately 55 species (Jin et al., 2012; Yu, Zhang, Shao, et al., 2015). Lily bulb is rich in many nutrients and bioactive substances, including protein, starch, dietary fiber, polyphenols, saponin, and vitamins. As a traditional medicine and food dual‐use resources, lily has also been processed into lily porridge, lily vermicelli, lily noodles, lily cocktail, and other foods as well as some antioxidant products (Yu et al., 2015).

Starch is the main component of lily bulbs, accounting for 53%–69% of dry weight (Li et al., 2019). Lily starch and other natural starch can be used as thickener, gelling agent, colloidal stabilizer, and filler (Song et al., 2017). However, the disadvantages of natural starch, such as easy aging, gelatinization, low solubility in cold water, poor heat resistance, poor shear force, and poor mechanical resistance and structural change, limit its application in the food industry. Therefore, various physical, chemical, and biological methods have been used to change the structure of starch granules and endow its characteristic properties (Li et al., 2014; Piecyk et al., 2018). In the previous study, our laboratory modified lily starch by using heat–moisture and acid treatments and found that modified lily starches presented enhanced physicochemical properties and could be used in industrial‐resistant starch and food ingredients (Li et al., 2020). Starch depolymerization easily occurred in the acid treatment and was unconducive to the environment. Therefore, an environment‐friendly modification method for lily starch should be developed.

Ultra‐high pressure (UHP) treatment is a typical physical nonthermal modification method. It is regarded as a green and environment‐friendly physical modification method and can be used for starch modification. In previous reports, the effects of UHP on different kinds of starch have been studied. Li et al. (2015) found that UHP treatment could promote water molecules to enter red adzuki bean starch granules, disrupt the crystalline structure, and reduce the thermal stability; Guo, Zeng, Lu, et al. (2015) found that UHP‐treated lotus seed starch showed lower retrogradation tendency compared to native starch; Larrea‐Wachtendorff et al. (2019) obtained high viscosity and highly structured potato starch hydrogels through UHP‐treated. Alvarez et al. (2014) found that the chickpea flour slurry product would exist better flow characteristics after modified by UHP‐treated. Liu et al. (2018) found that the in vitro digestibility of pea starch treated with UHP was remarkable lower than that of the native starch. These studies have indicated that UHP treatment could change the structure and physicochemical properties of starch and endow starch with new functional properties. Different crystallite types of starch show diverse results after UHP treatment. A‐type starch is the most sensitive to pressure, followed by C‐ and B‐type starch (Kim et al., 2018). Sorghum (A‐type) is completely gelatinized at 480–600 MPa (Liu et al., 2016). The starch of mung bean (C‐type) is completely gelatinized at 600 MPa (Li et al., 2011). Potato starch (B‐type) is gelatinized at 800 MPa (Błaszczak et al., 2005). The gelation degree of starch increased with the increases in water content and temperature. Although the pressure is adequately high, it could also cause complete gelation of starch at room temperature (Li et al., 2015).

However, to the best of our knowledge, no report is available on the physicochemical and morphological properties of lily starch treated with UHP. In this study, lily starch was treated at six UHP levels. The surface morphology, particle size, X‐ray patterns, thermal properties, Fourier‐transform infrared (FTIR) spectrum, and pasting properties of native lily and UHP‐treated starch were investigated. The experimental results can provide references for the application of lily starch.

## 2 MATERIALS AND METHODS

### 2.1 Materials

The fresh bulbs (*Lilium brownii var. viridulum* Baker) used in this work were cultivated in Longhui County, Hunan Province, China. After harvesting, the bulbs were transported to the laboratory immediately. Bulbs with physical damage and pests were selected out. Undamaged lily bulbs were peeled and washed with tap water, and then, the washed lily scales were placed into a wall breaker to homogenate for 2 min (v_lily scales_: v_dstilled water_ = 1:2).

### 2.2 Lily starch isolation

The lily bulb starch was extracted according to Zhang, Saleh, et al. (2020) with some modifications. The well‐homogenized lily slurry was filtered with a 100 mesh nylon cloth, and the residue was rinsed repeatedly with distilled water until no more starch filtrate was released. The collected filtrate was centrifuged at 25°C at 5,000 × g for 10 min. The precipitated starch granules were then washed with 0.05 mol/L of NaOH and stirred every 30 min. The NaOH solution was replaced every 3 hr until the supernatant of the cleaning solution became colorless and the cleaning was stopped. The starch samples were freeze‐dried, sifted through 100 mesh, and stored in a desiccator for further use.

### 2.3 UHP treatment

Lily starch was subjected to UHP treatment (high‐pressure press‐type SHPP‐8.8 L, Shanxi Sanshuihe Technology Co., Ltd.). The extracted starch was prepared into 15% (w/w) starch–water suspension and divided into seven equal parts. The suspension was packed into polythene bags, shaken thoroughly to move the bubbles of the vacuum bag, and sealed with a vacuum packer. The sealed samples were transferred into a pressure chamber and subjected to different pressure levels (100, 200, 300, 400, 500, and 600 MPa) at room temperature for 30 min. After UHP treatment, the samples were vacuum‐filtered, and the supernatant was removed after centrifugation with 5,000 × *g* at 25°C for 10 min. The samples were freeze‐dried, and each dried starch sample was pulverized with pestle and mortar and then kept in a desiccator at room temperature for further analysis.

### 2.4 Scanning electron microscopy

Scanning electron microscopy (SEM) of lily starch samples was obtained on a scanning electron microscope (EVO LS10, Carl Zeiss) following the method of Ovando‐Martínez et al. (2011). Approximately 1 mg of the native starch or UHP‐treated starch samples was sprinkled on a double‐sided adhesive tape, mounted on an aluminum stub and coated with gold for 30 s (25 mA, 2 × 10^−4^ MPa). Scanning electron micrographs were taken at 500 magnifications.

### 2.5 Polarized light microscopy (PLM)

Polarized and normal light microscopic images were recorded on a polarized light microscope (LEICA DM4500P; Leica Microsystems) by using the method of Guo, Zeng, Zhang, et al. (2015). A starch sample of 1 mg was dispersed in glass slides with glycerol and water (1:1, v/v). The PLM instrument was used to observe at 200× magnification under normal and polarized light conditions.

### 2.6 Particle size determination

The particle size parameters of starch were measured using a laser diffraction particle size analyzer (LS‐POP laser particle size analyzer, Omec Technology Co., Ltd.), as described by Li et al. (2019). In detail, 1.5 g of lily starch was evenly dispersed with 20 ml of distilled water, and then, the suspension was poured into the rotating container of the diffraction particle size analyzer with a shading ratio ranging from 8% to 15%. Refractive indices (dn/dc) of starch and water were set to 1.60 and 1.33, respectively. Volume particle size (*D*_(4,3)_), surface particle size (*D*_(3,2)_), *D*_10_, *D*_50,_ and *D*_90_ were recorded. *D*_(4, 3)_ presents the particle diameter of volume, *D*_(3, 2)_ presents the particle diameter of surface, and *D*_10_, *D*_50_, and *D*_90_ represent the corresponding particle sizes which are smaller than 10%, 50%, and 90% of the sample particles, respectively.

### 2.7 X‐ray diffraction analysis

X‐ray diffractograms of lily starch samples were obtained using an X‐ray diffractometer (XRD‐6000, Shimadzu) in accordance with the method of Ahmed et al. (2018) with some modifications. The measurement was operated at Cu‐Kα (*λ* = 1.5418 nm), X‐ray tube 40 kV, and voltage of 40 mA, the diffraction scanning angle was from 5° to 45° (2*θ*), the step size was 0.015°, and the scanning speed was 8°/min. The relative crystallinity of lily starch was calculated using MDI Jade software.

### 2.8 FTIR spectroscopy

The FTIR spectra of starch samples were recorded on an FTIR spectrophotometer (Model IRAffinity−1, Shimadzu) at room temperature, as described by Rafiq et al. (2016). Starch was mixed with dried KBr powder in a ratio of 1:100 (m/m) and pressed into transparent tablets under infrared light before measurement. KBr was scanned as background, and the spectra were recorded within the range of 400–4,000 cm^−1^.

### 2.9 Determination of thermal properties

The thermal properties of the lily starch samples were measured using a differential scanning calorimeter (Q2000‐DSC, TA Instruments) in accordance with the method of Li et al. (2020). Each starch sample of 5.0 mg was accurately weighed into a differential scanning calorimetry (DSC) pan, and 10 μL of distilled water was added. Before the experiment, the samples were balanced at room temperature for 24 hr, with an empty aluminum pan as a reference. The scanning temperature was from 30°C to 110°C, and the heating rate was 10°C/min. The thermal parameters, including onset temperature (*T*_o_), peak temperature (*T*_p_) and conclusion temperature (*T*_c_), the gelatinization temperature range (Δ*T*_r_), and the gelatinization enthalpy (Δ*H*), were recorded.

### 2.10 Pasting properties

The test was performed as described by Zhang, Ma, et al. (2020) with some modifications. The pasting properties were analyzed using a rapid viscoanalyzer (RVA Super‐4, Newport Scientific). Each sample (3.0 g, dry basis) was weighed into an RVA canister and added with 25 ml of distilled water. The slurry was then homogenized using a plastic paddle to avoid lump formation before the RVA run. The starch slurry was heated from 50°C to 95°C at 12°C/min and kept at 95°C for 2.5 min, then it was cooled to 50°C at the same rate with a paddle speed of 160 rpm. Pasting properties, including peak viscosity (PV), trough viscosity (TV), breakdown (BD), final viscosity (FV), setback (SB), peak time (PT), and pasting temperature (PT), were determined.

### 2.11 Statistical analysis

All the tests were performed in triplicate. Data were expressed as mean ± standard deviations. Statistical analysis was conducted using SPSS20.0 for Windows, and data were analyzed using ANOVA with Duncan's multiple range tests (*p* < .05). ORIGIN 7.5 was also used for statistical analysis.

## 3 RESULTS AND DISCUSSION

### 3.1 Morphological properties

**Figure 1:**
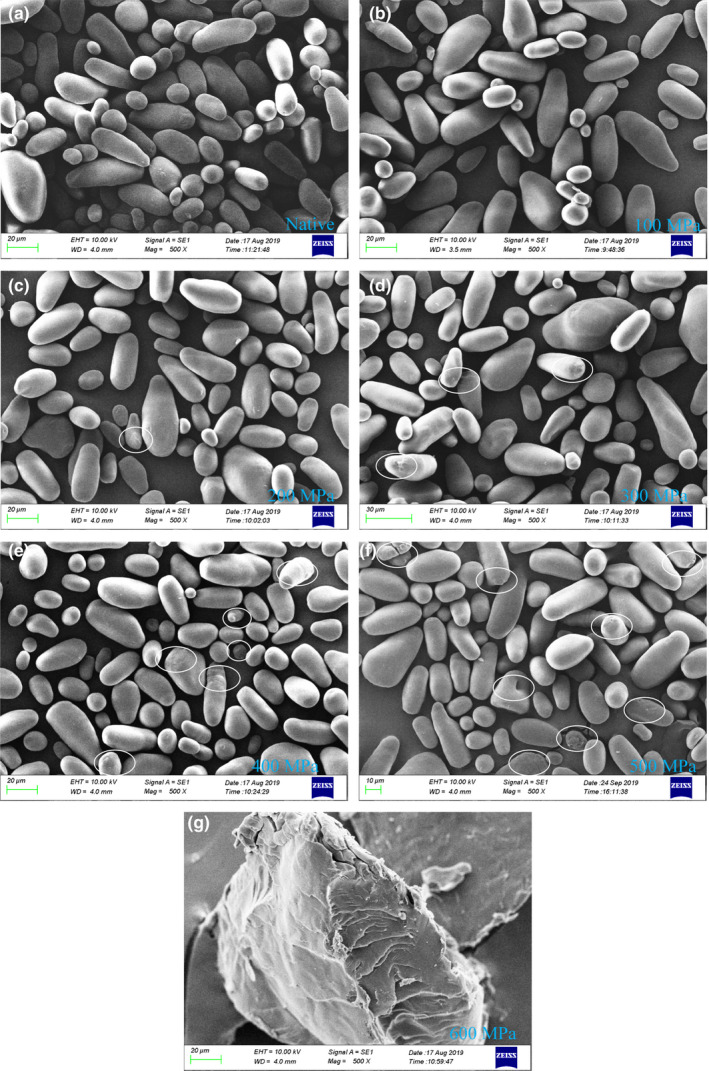
SEM images of the native and ultra‐high pressure treated starch granules at ×500 magnification

The microscopic images of the native and UHP‐treated lily starches with SEM are shown in Figure 1 (500×). Native lily starches showed round‐shaped, oval‐shaped, and other irregular granules with a smooth surface; this result is similar to a previous result observed by Li et al. (2020). After UHP treatment with the pressure ranging from 100 MPa to 400 MPa, the appearance of starch granules did not change significantly, which implied that the starch structure cannot be disrupted under these pressures (Figure 1b–e). When the treatment pressure was further increased to 500 MPa, the surface of starch granules began to shrink, and starch granules presented some fragments and a tendency to expand (Figure 1f). These observations demonstrated that the structure of the lily starch granules started to lose when the treatment pressure was increased to 500 MPa. The structure of starch granules was almost entirely disrupted, and a gel‐like appearance was observed at 600 MPa (Figure 1g). Lily starch had been nearly complete gelatinized at 600 MPa. This observation could be ascribed to the gelatinization of the lily starches at 600 MPa; previous reports have proven that UHP treatment with sufficiently high pressure could induce the gelatinization of quinoa starch (Ahmed et al., 2018), chickpea starch (Alvarez et al., 2014), and waxy wheat starch (Hu et al., 2017).

### 3.2 Light microscopy

**Figure 2:**
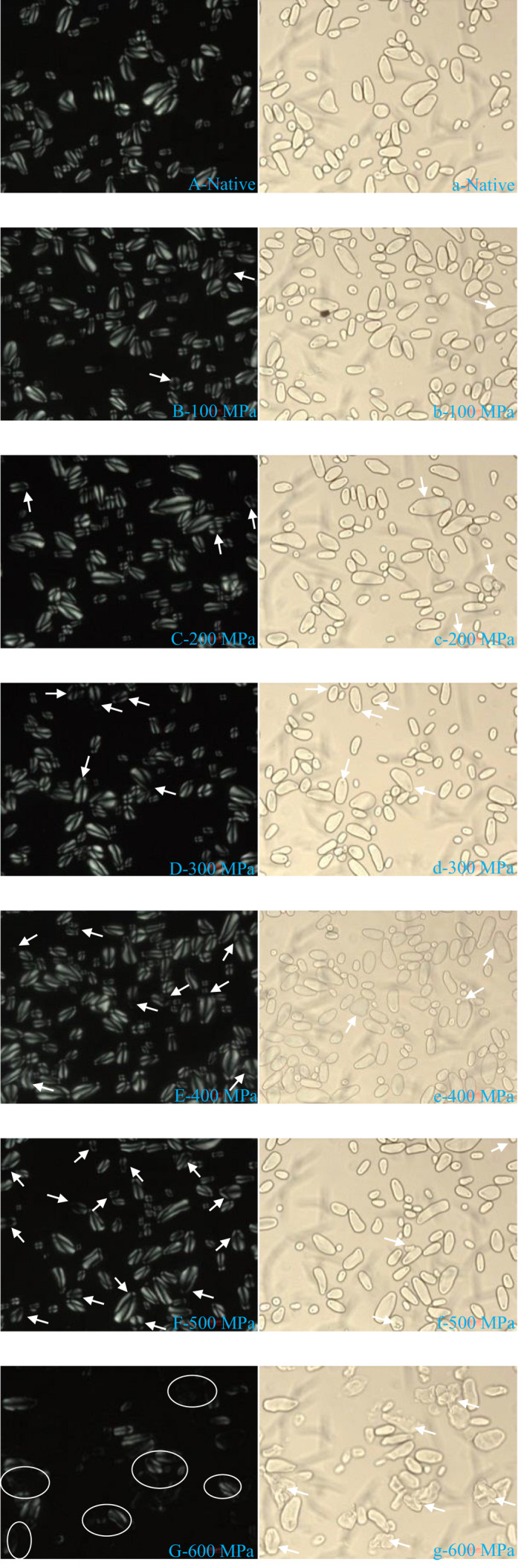
Morphological characteristics of the native and ultra‐high pressure treated starch granules under normal light × 200 (NLM), and polarized light × 200 (PLM)

The birefringence phenomenon of the native and UHP‐treated starch granules was observed under polarized and normal light. The results are shown in Figure 2. The size and shape of starch observed in natural light were basically the same as those observed in SEM, all the lily native starch presented a typical birefringence pattern, and the Maltese cross was evident under PLM. The umbilical points and grain morphology of native starch granules can be obviously observed under normal light. A similar phenomenon has been observed in winter wheat starch granules (Li et al., 2013). No significant difference in starch birefringence pattern existed between native starch granules and UHP‐treated ones at the pressure level of 100–400 MPa. The results showed that a few particles lost umbilical points and birefringence among the above pressure levels (Figure 2B–E and b–e). However, after treatment at 500 MPa, substantial lily starch granules lost their birefringence, and the polarization cross became unclear compared with the native starch granules (Figure 2F and f). At 600 MPa, almost all of the starch granules lost the Maltese cross observed via polarized light microscopy. Almost all the granules were disrupted (Figure 2G and g), and their umbilical points could not be observed under natural light, indicating that starch had been nearly complete gelatinized under this pressure. Starch granules were nonthermally gelatinized and lost their “Maltese cross” under a critical pressure level, which varied depending on the botanical source of starch. The critical pressure of rice starch (Li et al., 2012) and cassava starch (Liu et al., 2012) was 600 MPa, that of barley starch was 550 MPa (Stolt et al., 2000), and that of glutinous corn starch (Liu et al., 2012) was 450 MPa. The mechanism of UHP affecting starch polarization crossing is unclear. Nevertheless, some researchers have supposed that the double helix of amylopectin is disrupted as pressure increases, which causes the loss of polarization cross (Błaszczak et al., 2005). From the experimental results, the birefringence of lily starch nearly complete disappeared under the treatment of 600 MPa. SEM results also showed that the crystal structure of the particles was seriously damaged. This condition indicated that 600 MPa was the critical pressure for the complete gelation of lily starch.

### 3.3 3 Particle size distribution (PSD)

**Figure 3:**
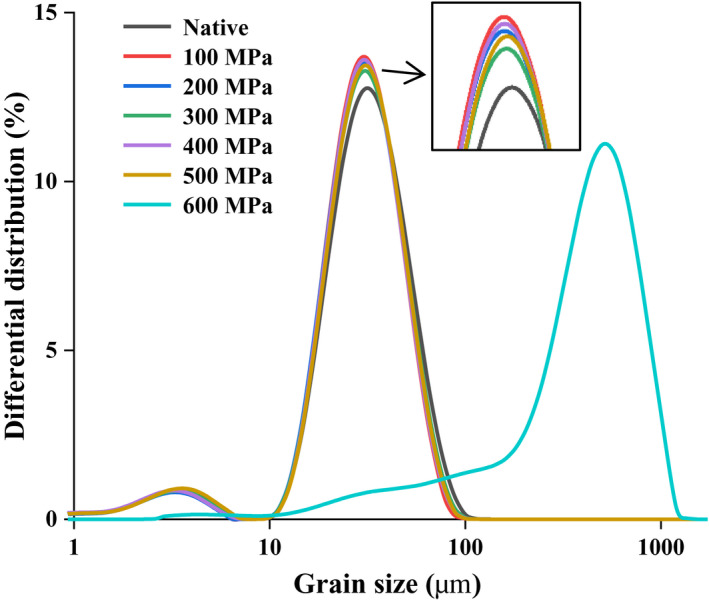
The particle size distribution of the native and ultra‐high pressure treated lily starch granules

The PSD of the native and UHP‐treated starch is shown in Figure 3. The grain size of lily starch showed two distribution peaks when the starch was treated at 0–500 MPa, and the maximum distribution peaks were approximately 3.55 and 31.16 μm. However, when the pressure reached 600 MPa, the PSD of starch decreased from two peaks to one peak, the PSD range was narrowed, and the maximum distribution peak was approximately 516.82 μm. This result is consistent with the result of 600 MPa starch granules bound tightly observed via SEM. Wei et al. (2016) proposed that UHP would disrupt starch granules and cause their size distribution to be within a certain range. The PSD parameter of native and UHP‐treated starch is presented in Table 1. *D*_(4, 3)_, *D*_(3, 2)_, *D*_10_, *D*_50,_ and *D*_90_ for 0–500 MPa high‐pressure treatment were 31.24–32.25 μm, 16.72–17.68 μm, 15.58–15.95 μm, 29.57–30.36 μm, and 50.59–52.96 μm, respectively. Such parameters for 600 MPa treatment were 80.40, 429.45, 789.24, 137.79, and 439.98 μm, respectively. No obvious difference existed in the size of starch granules treated with 0–500 MPa, but the size of starch granules treated with 600 MPa increased significantly; this result is consistent with the observation results of SEM and PNM. The gelation of starch granules resulted in the expansion and aggregation of starch granules, which led to an increase in starch particle size. A similar phenomenon has been observed for quinoa starch (Zhu & Li, 2019), pea starch (Liu et al., 2018), and tartary buckwheat starch (Liu, Guo, et al., 2016). The results showed that the effect of UHP on the morphology of starch granules was related to the pressure, with 600 MPa resulting in a transition of the lily native starch structure to gelatinized starch paste.

**Table 1:** Characteristic of the particle size distribution of the native and ultra‐high pressure treated lily starch granules

Pressure	*D*_10_ (μm)	*D*_50_ (μm)	*D*_90_ (μm)	*D*_(3,2)_ (μm)	*D*_(4,3)_ (μm)
Native	15.87 ± 0.06^a^	30.36 ± 0.14^c^	52.96 ± 0.40^a^	17.20 ± 0.08^ab^	32.25 ± 0.17^b^
100 MPa	15.94 ± 0.09^a^	29.57 ± 0.25^a^	50.59 ± 0.71^a^	17.57 ± 0.14^b^	31.24 ± 0.39^a^
200 MPa	15.95 ± 0.07^a^	29.70 ± 0.21^ab^	51.27 ± 0.52^a^	17.68 ± 0.10^b^	31.55 ± 0.27^ab^
300 MPa	15.78 ± 0.08^a^	30.02 ± 0.22^bc^	52.30 ± 0.67^a^	17.35 ± 0.12^ab^	31.94 ± 0.29^ab^
400 MPa	15.77 ± 0.06^a^	29.69 ± 0.13^ab^	51.07 ± 0.33^a^	16.72 ± 0.08^a^	31.41 ± 0.18^a^
500 MPa	15.58 ± 0.07^a^	29.97 ± 0.17^abc^	51.64 ± 0.41^a^	17.08 ± 0.09^ab^	31.67 ± 0.20^ab^
600 MPa	80.40 ± 0.91^b^	429.45 ± 0.21^d^	789.24 ± 2.95^b^	137.79 ± 0.83^c^	439.98 ± 0.74^c^

Note

Means followed by the same small letter within a column are not significantly different (*p* < .05); *D*_(4, 3)_ presents particle diameter of volume; *D*_(3, 2)_ presents particle diameter of surface; *D*_10_, *D*_50_, and *D*_90_ represent the corresponding particle size which is smaller than 10%, 50%, and 90% of the sample particles, respectively.

### 3.4 X‐ray diffraction

**Figure 4:**
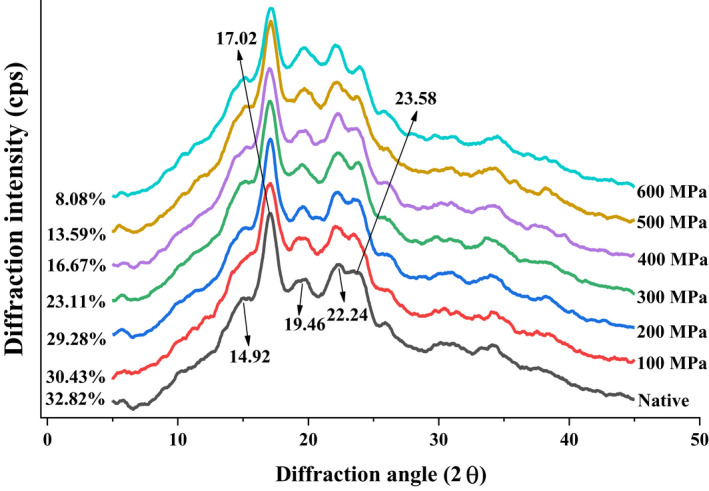
X‐ray diffraction spectra of native and ultra‐high pressure treated lily starch

In accordance with the X‐ray diffraction pattern, the starch can be divided into three categories: A‐type (mainly exists in corn starch, with a strong diffraction peak at 2*θ* values of 15°, 17°, 18°, and 23°), B‐type (mainly exists in the tubers of plants rich in carbohydrates, with a very strong diffraction peak at 17° and weak diffraction peaks at 20°, 22°, and 24°), and C‐type (mainly exists in leguminous plants; it is a mixture of A‐ and B‐type starch) (Liu, Guo, et al., 2016; Zobel, 1988). The native starch presented diffraction peaks at 14.92°, 17.02°, 19.46°, 22.24°, and 23.58° (Figure 4). The diffraction peak at 17.02° was very strong; hence, lily starch is a typical B‐type starch. After the UHP treatment from 100 to 600 MPa, no changes in X‐ray diffraction patterns could be observed, the disappearance of characteristic diffraction peak was not found, the change in crystal properties was mainly manifested as the change in diffraction peak strength, and the crystal type was still B‐type. The relative crystallinity of lily native starch was 32.82%. The relative crystallinity of starch was 30.43%, 29.28%, 23.11%, 16.67%, 13.59%, and 8.08% after UHP treatment from 100 to 600 MPa, indicating that UHP treatment could reduce the crystallinity of starch. This condition also suggested that the crystalline structure of starch was vulnerable to disruption at high pressure levels. In the previous research, potato starch also maintained a B‐type diffraction pattern after UHP treatment (Błaszczak et al., 2005; McPherson & Jane, 1999) Sorghum (A‐type) and mung bean starch (C‐type) were completely gelatinized at 600 MPa, and X‐rays showed that the starch granules gradually exhibited the diffraction pattern of B‐type crystal (Liu et al., 2016; Li et al., 2011). The B‐type starch was insensitive to UHP compared with A‐ and C‐type starch because it had a more open structure containing a hydrated helix core (Liu et al., 2010).

### 3.5 Thermal properties

**Figure 5:**
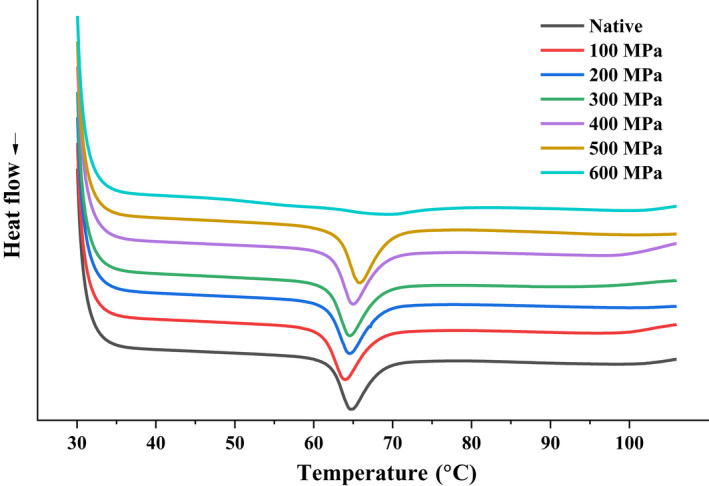
Differential scanning calorimetry thermograms of the native starch and ultra‐high pressure treated lily starch

Figure 5 shows the DSC diagram of the native and different UHP‐treated lily starch granules. With increasing pressure, the endothermic peak was gradually shifted to an increased temperature and became weak. A similar phenomenon has been observed for lotus seed starch (Guo, Zeng, Lu, et al., 2015). No thermal parameters existed for the lily starch treated at 600 MPa, and the endothermic peak of 600 MPa curve was indeed not so smooth, with tiny peaks appearing. Suggesting that UHP treatment at 600 MPa resulted in nearly complete gelatinization. The crystalline structure and molecular order of lily starch granules messed up. The thermal characteristics of native and lily starch granules treated using different UHP levels are summarized in Table 2. ∆*T*_r_ and ∆H for 0–500 MPa high‐pressure treatment were 7.05°C–7.69°C and 11.62–12.16 J/g, respectively. The parameters for 600 MPa treatment were not detected. The UHP treatment at 500 MPa caused decreased onset temperature and enthalpy of gelatinization compared with the treatment at 400 and 600 MPa. This result suggested that the samples treated at 500 MPa had better crystal structure than those treated at 400 and 600 MPa. The starch crystal structure might have undergone an annealing stage during UHP treatment. Consequently, the crystalline region was further strengthened, while the amorphous region was disrupted. Two types of phase transitions, namely, helix–helix dissociation and helix–coil transition, occurred in starch granules under different treatment conditions (Liu, et al., 2009; Liu, Yu, et al., 2009; Zeng et al., 2018). Relatively low pressure could not disrupt the double helices to coil state, the helices could only be separated side by side, and the helices could be dissociated into a single helix only when a certain pressure was reached. The pressure treatment of starch below 500 MPa disturbed the crystal structure of the lily starch, and 600 MPa was the critical pressure to disrupt the crystal structure. Pressure is an important factor in starch gelatinization.

**Table 2:** Thermal characteristics of the native and ultra‐high pressure treated lily starch granules

Pressure	DSC parameters				
	*T*_0_ (°C)	*T*_p_ (°C)	*T*_c_ (°C)	∆*T*_r_ (°C)	∆*H* (J/g)
Native	62.11 ± 0.06^a^	64.57 ± 0.01^b^	69.36 ± 0.01^a^	7.25 ± 0.01^bc^	11.63 ± 0.06^a^
100 MPa	61.10 ± 0.07^b^	63.84 ± 0.06^a^	68.80 ± 0.02^b^	7.68 ± 0.02^d^	11.72 ± 0.18^abc^
200 MPa	61.64 ± 0.09^c^	64.57 ± 0.09^b^	69.33 ± 0.01^c^	7.69 ± 0.01^d^	11.92 ± 0.16^bcd^
300 MPa	61.90 ± 0.09^a^	64.64 ± 0.23^b^	69.01 ± 0.01^d^	7.20 ± 0.14^c^	11.62 ± 0.19^ab^
400 MPa	62.37 ± 0.06^d^	64.96 ± 0.05^c^	69.42 ± 0.01^e^	7.05 ± 0.01^b^	12.16 ± 0.11^d^
500 MPa	63.29 ± 0.13^e^	65.85 ± 0.09^d^	69.62 ± 0.01^f^	6.33 ± 0.01^a^	11.98 ± 0.06^cd^
600 MPa	ND	ND	ND	ND	ND

Note

Means followed by same small letter within a column are not significantly different (*p* < .05).

Abbreviations: ND, not detected; *T*_c_, conclusion temperature; *T*_o_, onset temperature; *T*_p_, peak temperature; Δ*H*, enthalpy of gelatinization; Δ*T*_r_, gelatinization temperature range (Δ*T*_r_ = *T*_c_–*T*_o_).

### 3.6 FTIR spectroscopy

**Figure 6:**
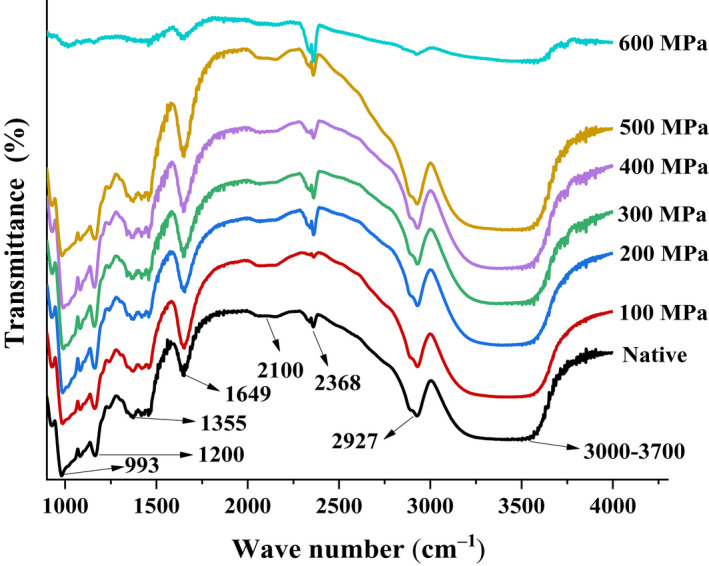
Fourier‐transform infrared spectra of the native and ultra‐high pressure treated lily starch

Fourier‐transform infrared spectroscopy of native and UHP‐treated starches is presented in Figure 6. No new peaks were found in the FTIR spectra after the UHP treatment compared with the native starch. The peak intensity varied among different bands, indicating that no new substances were produced but some molecular structures were changed. The sharp peak at approximately 993 cm^−1^ observed in the lily starch samples corresponded to the crystallinity of the starches (Remya et al., 2018). However, the peak weakened when the pressure reached 600 MPa, suggesting that the 600 MPa treatment significantly reduced the crystallinity of lily starch. The band at approximately 1,200–1,355 cm^−1^ was caused by the C–H bending or stretching vibration of carbohydrates (Zheng & Li, 2018). The reduction in bands in the 1,200–1,355 cm^–1^ region indicated the breakage of C–H bonds after the 600 MPa treatment. The sharp band at 1,649 cm^–1^ corresponded to the molecules absorbed in the amorphous region and the stretching vibration of the amide C = O band; the peak at 2,100 cm^−1^ originated from the free moisture content (Dankar et al., 2018). The intensity of peak at 2,100 cm^−1^ increased with UHP treatment at 0–500 MPa, thereby indicating that the amount of free water in the above starches increased. The peak was relatively gentle at 600 MPa, indicating that the content of free water in starch decreased significantly. The peak at 2,368 cm^−1^ was due to C–H stretching associated with ring methane hydrogen atoms (Rafiq et al., 2016). Substantial hydroxyl groups exist in natural starch, which induce intermolecular and intramolecular hydrogen bonds in the main chain of starch. The double‐helix structure was held together by hydrogen bonds. However, in the gelation process, water penetration disrupted this structure, resulting in infrared spectral fluctuations in the relevant region. The bond at 2,927 cm^−1^ was related to the C–H stretching and bending vibration of methyl and methylene groups of polysaccharides (Wang, Xie, et al., 2020; Weerapoprasit & Prachayawarakorn, 2019). The peak intensity weakened after 600 MPa treatment, implying that the content of polysaccharides reduced after UHP treatment. The absorption bands in the 3000–3700 cm^–1^ region were related to the absorption of hydrogen‐bonded O–H groups in starch (Kizil et al., 2002; Wang et al., 2020). The widening of this range after UHP treatment indicated that the starch molecule bond was broken by hydrogen bond, exposing considerable O–H.

### 3.7 Pasting properties

**Figure 7:**
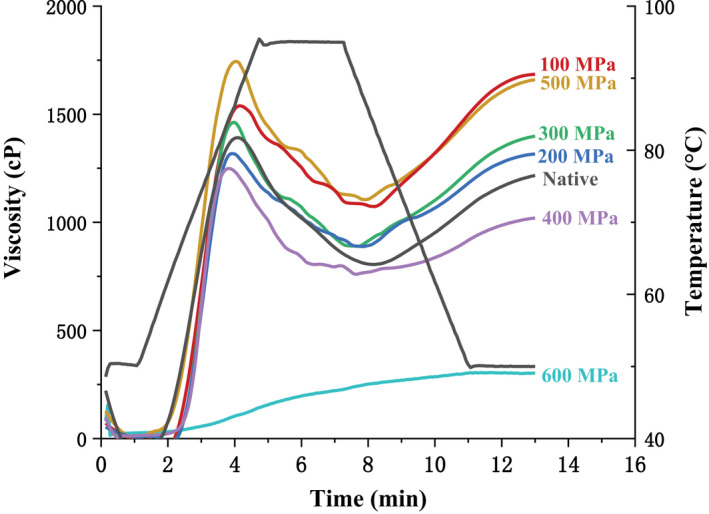
Pasting properties of the native starch and ultra‐high pressure treated lily starch

Variations in pasting properties of the native and UHP‐treated samples are shown in Table 3 and Figure 7. From the table, the PV, TV, FV, and SB of starch treated using UHP of 100–500 MPa all showed an upward trend, whereas BD and SB showed opposite results. Lily starch treated at 600 MPa exhibited the lowest PV, TV, BD, FV, and SB values, PT value was the highest, and GT was not detected. Previous studies have shown that the change in each parameter is caused by the change in grain structure during the transformation of starch crystal structure (Hu et al., 2011; Li et al., 2011). PV was related to the expansion and hydration of starch particles in the early stage. The SB value of lily starch under 100–500 MPa pressure was significantly higher than that of the original starch, indicating a higher retrograde tendency. The experimental results showed that high pressure below 600 MPa could promote the expansion and gelatinization of starch, whereas starch was almost completely gelatinized at 600 MPa. The reduction in starch swelling could cause a decrease in BD, and the stability of starch paste could be enhanced, which would lead to an increase in starch expansion temperature. Hu et al. (2011) showed that water would enter starch, cause internal hydrogen bonds to break, resulting in instability in the crystalline and amorphous regions, and gelatinize starch when it is treated under high pressure. The change trend of the pasting properties of acorn kernel starch treated using UHP is consistent with this experiment (Li et al., 2018).

**Table 3:** Pasting properties of the native starch and ultra‐high pressure treated lily starch

Pressure	PV (cp)	TV (cp)	BD (cp)	FV (cp)	SB (cp)	PT (min)	GT (°C)
Native	1,409 ± 84^b^	801 ± 37^cb^	607 ± 50^bc^	1,246 ± 55^ac^	445 ± 23^c^	3.71 ± 0.02^c^	66.10 ± 0.69^b^
100 MPa	1,694 ± 74^a^	982 ± 26^a^	711 ± 43^ab^	3,423 ± 10^c^	718 ± 21^a^	4.27 ± 0.44^b^	68.25 ± 0.76^a^
200 MPa	1,315 ± 45^b^	860 ± 21^b^	455 ± 66^c^	1,316 ± 16^bc^	456 ± 20^c^	3.93 ± 0.00^bc^	68.50 ± 0.02^a^
300 MPa	1,454 ± 97^b^	824 ± 48^cb^	629 ± 39^abc^	1,398 ± 25^b^	573 ± 18^b^	3.93 ± 0.05^bc^	68.75 ± 0.94^a^
400 MPa	1,251 ± 16^b^	784 ± 6^c^	503 ± 15^bc^	1,019 ± 39^d^	271 ± 33^d^	3.80 ± 0.05^bc^	67.30 ± 0.41^ba^
500 MPa	1,811 ± 105^a^	983 ± 12^a^	827 ± 117^a^	1,659 ± 53^a^	675 ± 40^a^	3.86 ± 0.06^bc^	63.95 ± 0.47^c^
600 MPa	221 ± 28^c^	199 ± 36^d^	22 ± 8^d^	303 ± 24^e^	104 ± 12^e^	6.95 ± 0.03^a^	ND

Note

Means followed by the same small letter within a column are not significantly different (*p* < .05).

Abbreviations: BD, breakdown; FV, final viscosity; GT, pasting temperature; ND, not detected.; PT, peak time; PV, peak viscosity; SB, setback; TV, trough viscosity.

## 4 CONCLUSIONS

In this article, lily starch was physically modified using UHP. UHP treatment showed an evident effect on the granule structural, PSD, and physicochemical properties of lily starch. The smooth lily starch surface became rougher and more agglomerate than before after UHP treatment. Lily starch treated at 600 MPa had the largest particle size and a distinct gelatinized structure. XRD results showed a decreased relative crystallinity after UHP treatment. The value of gelatinization temperatures gradually increased with increasing pressure. The lily starch treated at 600 MPa exhibited lower PV, TV, BD, FV, and SB compared with that treated at 0–500 MPa. The UHP treatment at 600 MPa for 30 min caused nearly complete gelatinization of lily starch. This experiment has a certain reference value for the nonheat treatment of lily starch. However, the exact mechanism of various parameter changes caused by UHP should be studied in the future.

## CONFLICT OF INTEREST

The authors declare no conflict of interest.

## Data Availability

The datasets used or analyzed during the current study are available from the corresponding author on reasonable request.

## References

[fsn32060-bib-0001] Ahmed, J. , Thomas, L. , Arfat, Y. A. , & Joseph, A. (2018). Rheological, structural and functional properties of high‐pressure treated quinoa starch in dispersions. Carbohydrate Polymers, 197, 649–657. 10.1016/j.carbpol.2018.05.081 30007658

[fsn32060-bib-0002] Alvarez, M. D. , Fuentes, R. , Olivares, M. D. , & Canet, W. (2014). Effects of high hydrostatic pressure on rheological and thermal properties of chickpea (*Cicer arietinum L*.) flour slurry and heat‐induced paste. Innovative Food Science & Emerging Technologies, 21, 12–23. 10.1016/j.ifset.2013.11.005

[fsn32060-bib-0003] Błaszczak, W. , Valverde, S. , & Fornal, J. (2005). Effect of high pressure on the structure of potato starch. Carbohydrate Polymers, 59(3), 377–383. 10.1016/j.carbpol.2004.10.008

[fsn32060-bib-0004] Dankar, I. , Haddarah, A. , Omar, F. E. , Pujolà, M. , & Sepulcre, F. (2018). Characterization of food additive‐potato starch complexes by FTIR and X‐ray diffraction. Food Chemistry, 260, 7–12. 10.1016/j.foodchem.2018.03.138 29699684

[fsn32060-bib-0005] Guo, Z. , Zeng, S. , Lu, X. , Zhou, M. , Zheng, M. , & Zheng, B. (2015). Structural and physicochemical properties of lotus seed starch treated with ultra‐high pressure. Food Chemistry, 186, 223–230. 10.1016/j.foodchem.2015.03.069 25976814

[fsn32060-bib-0006] Guo, Z. , Zeng, S. , Zhang, Y. , Lu, X. , Tian, Y. , & Zheng, B. (2015). The effects of ultra‐high pressure on the structural, rheological and retrogradation properties of lotus seed starch. Food Hydrocolloids, 44, 285–291. 10.1016/j.foodhyd.2014.09.014

[fsn32060-bib-0007] Hu, X. , Xu, X. , Jin, Z. , Tian, Y. , Bai, Y. , & Xie, Z. (2011). Retrogradation properties of rice starch gelatinized by heat and high hydrostatic pressure (HHP). Journal of Food Engineering, 106(3), 262–266. 10.1016/j.jfoodeng.2011.05.021

[fsn32060-bib-0008] Hu, X. P. , Zhang, B. , Jin, Z. Y. , Xu, X. M. , & Chen, H. Q. (2017). Effect of high hydrostatic pressure and retrogradation treatments on structural and physicochemical properties of waxy wheat starch. Food Chemistry, 232, 560–565. 10.1016/j.foodchem.2017.04.040 28490111

[fsn32060-bib-0009] Jin, L. , Zhang, Y. , Yan, L. , Guo, Y. , & Niu, L. (2012). Phenolic compounds and antioxidant activity of bulb extracts of six Lilium species native to China. Molecules, 17(8), 9361–9378. 10.3390/molecules17089361 22864243 PMC6269050

[fsn32060-bib-0010] Kim, S. , Yang, S. Y. , Chun, H. H. , & Song, K. B. (2018). High hydrostatic pressure processing for the preparation of buckwheat and tapioca starch films. Food Hydrocolloids, 81, 71–76. 10.1016/j.foodhyd.2018.02.039

[fsn32060-bib-0011] Kizil, R. , Irudayaraj, J. , & Seetharaman, K. (2002). Characterization of irradiated starches by using FT‐Raman and FTIR spectroscopy. Journal of Agricultural and Food Chemistry, 50(14), 3912–3918. 10.1021/jf011652p 12083858

[fsn32060-bib-0012] Larrea‐Wachtendorff, D. , Tabilo‐Munizaga, G. , & Ferrari, G. (2019). Potato starch hydrogels produced by high hydrostatic pressure (HHP): A first approach. Polymers, 11(10), 1673 10.3390/polym11101673 PMC6836192 31615036

[fsn32060-bib-0013] Li, H. , Wang, R. , Liu, J. , Zhang, Q. , Li, G. , Shan, Y. , & Ding, S. (2020). Effects of heat‐moisture and acid treatments on the structural, physicochemical, and in vitro digestibility properties of lily starch. International Journal of Biological Macromolecules, 148, 956–968. 10.1016/j.ijbiomac.2020.01.181 31972200

[fsn32060-bib-0014] Li, H. , Wang, R. , Zhang, Q. , Li, G. , Shan, Y. , & Ding, S. (2019). Morphological, structural, and physicochemical properties of starch isolated from different lily cultivars grown in China. International Journal of Food Properties, 22(1), 737–757. 10.1080/10942912.2019.1603998

[fsn32060-bib-0015] Li, W. , Bai, Y. , Mousaa, S. A. , Zhang, Q. , & Shen, Q. (2012). Effect of high hydrostatic pressure on physicochemical and structural properties of rice starch. Food and Bioprocess Technology, 5(6), 2233–2241. 10.1007/s11947-011-0542-6

[fsn32060-bib-0016] Li, W. , Cao, F. , Fan, J. , Ouyang, S. , Luo, Q. , Zheng, J. , & Zhang, G. (2014). Physically modified common buckwheat starch and their physicochemical and structural properties. Food Hydrocolloids, 40, 237–244. 10.1016/j.foodhyd.2014.03.012

[fsn32060-bib-0017] Li, W. , Gao, J. , Saleh, A. S. , Tian, X. , Wang, P. , Jiang, H. , & Zhang, G. (2018). The modifications in physicochemical and functional properties of proso millet starch after Ultra‐High pressure (UHP) process. Starch‐Stärke, 70(5–6), 1700235 10.1002/star.201700235

[fsn32060-bib-0018] Li, W. , Shan, Y. , Xiao, X. , Luo, Q. , Zheng, J. , Ouyang, S. , & Zhang, G. (2013). Physicochemical properties of A‐ and B‐starch granules isolated from hard red and soft red winter wheat. Journal of Agricultural and Food Chemistry, 61(26), 6477–6484. 10.1021/jf400943h 23756853

[fsn32060-bib-0019] Li, W. , Tian, X. , Liu, L. , Wang, P. , Wu, G. , Zheng, J. , & Zhang, G. (2015). High pressure induced gelatinization of red adzuki bean starch and its effects on starch physicochemical and structural properties. Food Hydrocolloids, 45, 132–139. 10.1016/j.foodhyd.2014.11.013

[fsn32060-bib-0020] Li, W. , Zhang, F. , Liu, P. , Bai, Y. , Gao, L. , & Shen, Q. (2011). Effect of high hydrostatic pressure on physicochemical, thermal and morphological properties of mung bean (*Vigna radiata L*.) starch. Journal of Food Engineering, 103(4), 388–393. 10.1016/j.jfoodeng.2010.11.008

[fsn32060-bib-0021] Liu, D. , Wu, Q. , Chen, H. , & Chang, P. R. (2009). Transitional properties of starch colloid with particle size reduction from micro‐to nanometer. Journal of Colloid and Interface Science, 339(1), 117–124. 10.1016/j.jcis.2009.07.035 19666174

[fsn32060-bib-0022] Liu, H. , Fan, H. , Cao, R. , Blanchard, C. , & Wang, M. (2016). Physicochemical properties and in vitro digestibility of sorghum starch altered by high hydrostatic pressure. International Journal of Biological Macromolecules, 92, 753–760. 10.1016/j.ijbiomac.2016.07.088 27477247

[fsn32060-bib-0023] Liu, H. , Guo, X. , Li, Y. , Li, H. , Fan, H. , & Wang, M. (2016). In vitro digestibility and changes in physicochemical and textural properties of tartary buckwheat starch under high hydrostatic pressure. Journal of Food Engineering, 189, 64–71. 10.1016/j.jfoodeng.2016.05.015

[fsn32060-bib-0024] Liu, H. , Yu, L. , Dean, K. , Simon, G. , Petinakis, E. , & Chen, L. (2009). Starch gelatinization under pressure studied by high pressure DSC. Carbohydrate polymers. Carbohydrate Polymers, 75(3), 395–400. 10.1016/j.carbpol.2008.07.034

[fsn32060-bib-0025] Liu, M. , Wu, N. N. , Yu, G. P. , Zhai, X. T. , Chen, X. , Zhang, M. , & Tan, B. (2018). Physicochemical properties, structural properties, and in vitro digestibility of pea starch treated with high hydrostatic pressure. Starch‐Stärke, 70(1–2), 1700082 10.1002/star.201700082

[fsn32060-bib-0026] Liu, P. , Hu, X. , & Shen, Q. (2010). Effect of high hydrostatic pressure on starches: A review. Starch‐Stärke, 62(12), 615–628. 10.1002/star.201000001

[fsn32060-bib-0027] Liu, P. , Zhang, Q. , Shen, Q. , Hu, X. , & Wu, J. (2012). Effect of high hydrostatic pressure on modified noncrystalline granular starch of starches with different granular type and amylase content. LWT, 47(2), 450–458. 10.1016/j.lwt.2012.02.005

[fsn32060-bib-0028] McPherson, A. E. , & Jane, J. L. (1999). Comparison of waxy potato with other root and tuber starches. Carbohydrate Polymers, 40(1), 57–70. 10.1016/S0144-8617(99)00039-9

[fsn32060-bib-0029] Ovando‐Martínez, M. , Osorio‐Díaz, P. , Whitney, K. , Bello‐Pérez, L. A. , & Simsek, S. (2011). Effect of the cooking on physicochemical and starch digestibility properties of two varieties of common bean (*Phaseolus vulgaris* L.) grown under different water regimes. Food Chemistry, 129(2), 358–365. 10.1016/j.foodchem.2011.04.084 30634238

[fsn32060-bib-0032] Piecyk, M. , Drużyńska, B. , Ołtarzewska, A. , Wołosiak, R. , Worobiej, E. , & Ostrowska‐Ligęza, E. (2018). Effect of hydrothermal modifications on properties and digestibility of grass pea starch. International Journal of Biological Macromolecules, 118, 2113–2120. 10.1016/j.ijbiomac.2018.07.063 30016659

[fsn32060-bib-0033] Rafiq, S. I. , Singh, S. , & Saxena, D. C. (2016). Effect of heat‐moisture and acid treatment on physicochemical, pasting, thermal and morphological properties of Horse Chestnut (*Aesculus indica*) starch. Food Hydrocolloids, 57, 103–113. 10.1016/j.foodhyd.2016.01.009

[fsn32060-bib-0034] Remya, R. , Jyothi, A. N. , & Sreekumar, J. (2018). Effect of chemical modification with citric acid on the physicochemical properties and resistant starch formation in different starches. Carbohydrate Polymers, 202, 29–38. 10.1016/j.carbpol.2018.08.128 30287003

[fsn32060-bib-0035] Song, M. R. , Choi, S. H. , Oh, S. M. , Kim, H. Y. , Bae, J. E. , Park, C. S. , & Baik, M. Y. (2017). Characterization of amorphous granular starches prepared by high hydrostatic pressure (HHP). Food Science and Biotechnology, 26(3), 671–678. 10.1007/s10068-017-0106-2 30263591 PMC6049584

[fsn32060-bib-0036] Stolt, M. , Oinonen, S. , & Autio, K. (2000). Effect of high pressure on the physical properties of barley starch. Innovative Food Science & Emerging Technologies, 1(3), 167–175. 10.1016/S1466-8564(00)00017-5

[fsn32060-bib-0037] Wang, C. , Xue, Y. , Yousaf, L. , Hu, J. , & Shen, Q. (2020). Effects of high hydrostatic pressure on the ordered structure including double helices and V‐type single helices of rice starch. International Journal of Biological Macromolecules, 144, 1034–1042. 10.1016/j.ijbiomac.2019.09.180 31669464

[fsn32060-bib-0038] Wang, Y. , Xie, Z. , Wu, Q. , Song, W. , Liu, L. , Wu, Y. , & Gong, Z. (2020). Preparation and characterization of carboxymethyl starch from cadmium‐contaminated rice. Food Chemistry, 308, 125674 10.1016/j.foodchem.2019.125674 31669944

[fsn32060-bib-0039] Weerapoprasit, C. , & Prachayawarakorn, J. (2019). Characterization and properties of biodegradable thermoplastic grafted starch films by different contents of methacrylic acid. International Journal of Biological Macromolecules, 123, 657–663. 10.1016/j.ijbiomac.2018.11.083 30445086

[fsn32060-bib-0040] Wei, B. , Cai, C. , Jin, Z. , & Tian, Y. (2016). High‐pressure homogenization induced degradation of amylopectin in a gelatinized state. Starch‐Stärke, 68(7–8), 734–741. 10.1002/star.201500250

[fsn32060-bib-0041] Yu, X. , Zhang, J. , Li, A. , Wang, Z. , & Xiong, F. (2015). Morphology and physicochemical properties of 3 Lilium bulb starches. Journal of Food Science, 80(8), C1661–C1669. 10.1111/1750-3841.12969 26194949

[fsn32060-bib-0042] Yu, X. , Zhang, J. , Shao, S. , Yu, H. , Xiong, F. , & Wang, Z. (2015). Morphological and physicochemical properties of bulb and bulbil starches from Lilium lancifolium. Starch‐Stärke, 67(5–6), 448–458. 10.1002/star.201400209

[fsn32060-bib-0043] Zeng, F. , Li, T. , Gao, Q. , Liu, B. , & Yu, S. (2018). Physicochemical properties and in vitro digestibility of high hydrostatic pressure treated waxy rice starch. International Journal of Biological Macromolecules, 120, 1030–1038. 10.1016/j.ijbiomac.2018.08.121 30171963

[fsn32060-bib-0044] Zhang, X. , Ma, Q. , Liu, X. , Zhang, D. , Ma, L. , Luo, D. L. , & Liu, X. (2020). Effect of microwave irradiation on the pasting, thermal, and rheological properties of cassava starch–sugar mixtures. Journal of Food Process Engineering, 43(8), e13431 10.1111/jfpe.13431

[fsn32060-bib-0045] Zhang, Z. , Saleh, A. S. M. , Wu, H. , Gou, M. , Liu, Y. U. , Jing, L. , Zhao, K. , Su, C. , Zhang, B. O. , & Li, W. (2020). Effect of Starch Isolation Method on Structural and Physicochemical Properties of Acorn Kernel Starch. Starch‐Stärke, 72(1–2), 1900122 10.1002/star.201900122

[fsn32060-bib-0046] Zheng, Y. , & Li, Y. (2018). Physicochemical and functional properties of coconut (*Cocos nucifera L*) cake dietary fibres: Effects of cellulase hydrolysis, acid treatment and particle size distribution. Food Chemistry, 257, 135–142. 10.1016/j.foodchem.2018.03.012 29622189

[fsn32060-bib-0047] Zhu, F. , & Li, H. (2019). Effect of high hydrostatic pressure on physicochemical properties of quinoa flour. LWT, 114, 108367 10.1016/j.lwt.2019.108367

[fsn32060-bib-0048] Zobel, H. F. (1988). Starch crystal transformations and their industrial importance. Starch – Stärke., 64(3), 1–7. 10.1002/star.19880400102

